# Advancing microbial ecology, microbiomes, and One Health in Africa: from regional initiatives to pan-African flagship programs

**DOI:** 10.1093/ismejo/wrag132

**Published:** 2026-05-21

**Authors:** Mohamed Hijri, Fatima Zahra Aliyat, Jean Legeay, Soon-Jae Lee, Mohamed Idbella, Aliya Fatima Anwar, Khaoula Errafii, Ramona Marasco, Manosh Kumar Biswas, Vittorio Venturi, Adolphe Zézé, MesfinTafesse Gemeda, Samuel C Eziuzor, Thulani Makhalanyane, Bulbul Ahmed

**Affiliations:** African Genome Center, Colleage of Agriculture and Environmental Science, University Mohammed VI Polytechnic (UM6P), Ben Guerir 43150, Morocco; Institut de Recherche en Biologie Végétale (IRBV), Département de Sciences Biologiques, Université de Montréal, Montréal, QC H1X 2B2, Canada; African Genome Center, Colleage of Agriculture and Environmental Science, University Mohammed VI Polytechnic (UM6P), Ben Guerir 43150, Morocco; African Genome Center, Colleage of Agriculture and Environmental Science, University Mohammed VI Polytechnic (UM6P), Ben Guerir 43150, Morocco; African Genome Center, Colleage of Agriculture and Environmental Science, University Mohammed VI Polytechnic (UM6P), Ben Guerir 43150, Morocco; Colleage of Agriculture and Environmental Science, University Mohammed VI Polytechnic (UM6P), Ben Guerir 43150, Morocco; African Genome Center, Colleage of Agriculture and Environmental Science, University Mohammed VI Polytechnic (UM6P), Ben Guerir 43150, Morocco; African Genome Center, Colleage of Agriculture and Environmental Science, University Mohammed VI Polytechnic (UM6P), Ben Guerir 43150, Morocco; Department of Biomedical Sciences, College of Health Sciences, Abu Dhabi University, Abu Dhabi, P.O. Box 59911, United Arab Emirates; Biological and Environmental Sciences and Engineering Division, King Abdullah University of Science and Technology, Thuwal 23955-6900, Saudi Arabia; African Genome Center, Colleage of Agriculture and Environmental Science, University Mohammed VI Polytechnic (UM6P), Ben Guerir 43150, Morocco; African Genome Center, Colleage of Agriculture and Environmental Science, University Mohammed VI Polytechnic (UM6P), Ben Guerir 43150, Morocco; International Centre for Genetic Engineering and Biotechnology (ICGEB), Trieste, Italy; Laboratoire de Microbiologie, Biotechnologies et Bioinformatique UMRI Sciences Agronomiques et Procédés de Transformation, Institut National Polytechnique Félix HOUPHOUËT-BOIGNY, Yamoussoukro BP 1093, Côte d’Ivoire; Department of Biotechnology, College of Natural and Applied Sciences, Addis Ababa Science and Technology University, Addis Ababa, P.O. Box 16417, Ethiopia; School of Engineering, University of British Columbia–Okanagan, Kelowna, BC V1V 1V7, Canada; Department of Microbiology, Faculty of Science, Stellenbosch University, Stellenbosch 7600, South Africa; African Genome Center, Colleage of Agriculture and Environmental Science, University Mohammed VI Polytechnic (UM6P), Ben Guerir 43150, Morocco

**Keywords:** Africa, ecosystems, microbiomes, microbial ecology, One Health

## Abstract

Microbial ecology and microbiome science are increasingly central to global “One Health” efforts, a framework that recognizes the interconnected health of humans, animals, and the environment. In Africa, these fields are particularly important for addressing interconnected challenges in public health, agriculture, and ecosystem resilience. Discussions at the ISME Africa Morocco 2025 regional meeting highlighted both progress and persistent gaps in African microbiome research. Initiatives such as the African BioGenome Project and Human Heredity and Health in Africa demonstrate the feasibility of population-representative studies, regional training, networking, and open-science frameworks; however, the research landscape remains fragmented, with limited intra-African collaboration and continued reliance on external funding and leadership. The development of Africa-specific reference genomes, population-based microbiome datasets, is essential to address these gaps and ensure global representation. This perspective synthesizes current evidence and outlines strategic priorities to transition from toward coordinated pan-African research networks and flagship programs. Key recommendations include developing Africa-specific genome reference datasets, establishing pan-continental consortia, fostering equitable African–non-African partnerships, integrating microbiome science into policy frameworks, and adopting FAIR open-science practices. Strengthening bioinformatics and computational capacity will be essential to transform fragmented data into high-impact, coordinated insights. Advancing these priorities will accelerate translation into One Health outcomes, including antimicrobial resistance surveillance, food security, climate-resilient agriculture, and precision medicine. Africa’s rich microbial diversity offers significant potential for antibiotic discovery, improved crop productivity, and sustainable agricultural systems. Collectively, strengthening Africa-led, collaborative microbiome research will enable the continent’s microbial diversity to drive impactful solutions with regional and global relevance.

## Introduction

**Figure 1:**
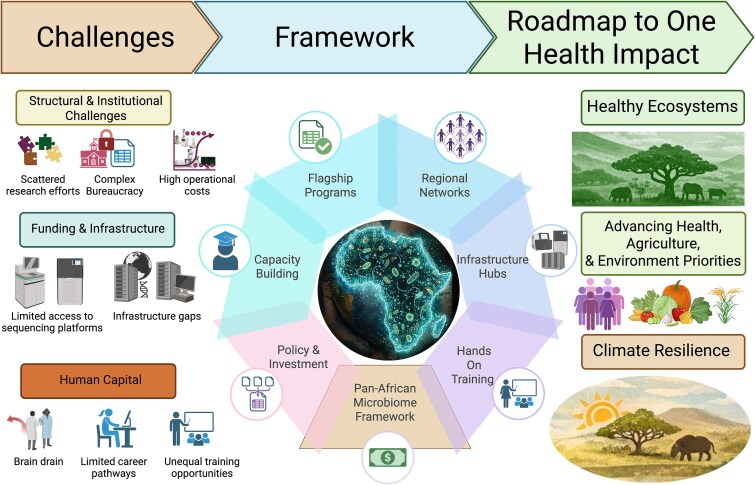
Conceptual overview of the challenges, framework, and roadmap toward One Health impact for microbiome research in Africa. The left panel highlights key structural and systemic constraints, including fragmented research efforts, limited funding, complex bureaucracy, inadequate infrastructure, weak regional networks, data and technology gaps, and brain drain.

Microbial ecology and microbiome science are at the forefront of transformative research in health, agriculture, and environmental sustainability. The One Health approach, which emphasizes the interdependence of human, animal, and environmental health, provides a critical framework for integrating these disciplines [1]. In Africa, the need for such integration is particularly acute due to the continent’s high burden of infectious and noncommunicable diseases, diverse agroecosystems, rapidly growing urban populations, and the impacts of climate change [2]. Despite this urgency, Africa remains substantially underrepresented in global microbiome initiatives, with most reference datasets derived by institutions and funding bodies outside the continent [2–4]. As a result, global microbiome projects often fail to capture Africa’s ecological, demographic, and cultural diversity, creating significant knowledge gaps in the understanding of microbial dynamics in local contexts [5, 6]. These gaps are not only regional but also have global implications, as the underrepresentation of African microbiomes limits the completeness and generalizability of microbiome science worldwide. Many studies conducted in Africa have been further limited by small sample sizes, narrow geographic scope, and inconsistent methodologies, constraining continent-wide comparisons and synthesis [2, 7] (Fig. 1).

Recent initiatives, including the African BioGenome Project (AfricaBP), which is designed to expand genomics research, infrastructure, and capacity across Africa with leadership, technologies, and data rooted primarily within the continent rather than externally driven, demonstrate the growing capacity of African institutions to conduct large-scale, population-representative studies. Together, these efforts signal a paradigm shift toward genomics and microbiome science led from Africa and aligned with African priorities. Projects such as AfricaBP [8–10] and the Africa Wits-INDEPTH Partnership for Genomic Research (AWI-Gen) 2 Microbiome Project have generated continent-specific datasets, trained hundreds of early-career scientists, and begun developing regional digital sequence repositories [6]. These initiatives underscore the feasibility of building continental-scale research platforms while addressing longstanding capacity gaps.

Challenges remain (Fig. 1). Research funding remains predominantly external, intra-African collaboration remains limited, and scientific leadership is often concentrated outside the continent [3]. Microbiome research across Africa is still constrained by both geographic coverage and local leadership: only 33 of 54 countries have published studies, and 80% of these are led by senior authors based outside Africa [4]. Such dynamics increase the risk that African participants will be treated as research subjects rather than equitable partners, thereby impeding sustainable capacity building and long-term development.

In this perspective, we synthesize current literature and propose a conceptual framework for African-led microbiome science that integrates One Health priorities with continental coordination, data sovereignty, and capacity development. Rather than providing a purely descriptive overview, we outline actionable ways for Africa to lead the next generation of global microbiome research. We propose a strategic framework for expanding microbial ecology and microbiome research across Africa, emphasizing flagship programs, pan-African research networks, capacity building, open science, and policy integration. Our goal is to provide a roadmap for transforming fragmented initiatives into a coordinated, continent-wide effort aligned with Africa’s health, agricultural, and environmental priorities (Fig. 1).

### ISME Africa 2: consolidating a pan-African platform for microbial ecology and capacity building

Building on the success of the first ISME Africa meeting held in Yamoussoukro, Côte d’Ivoire (22–26 May 2023), the second edition further consolidated a pan-African platform dedicated to advancing microbial ecology research and strengthening continental and international collaborations. ISME Africa 2 attracted 208 applications, of which 151 participants were selected, reflecting both high demand and a commitment to scientific excellence. Gender representation was well balanced (72 women and 79 men), highlighting encouraging progress toward inclusivity in the field. Participants represented 22 countries across Africa and beyond, reflecting broad geographic diversity and increasing international engagement.

The scientific program was structured around four major thematic tracks: (I) Human, Animal & One Health Microbiomes; (II) Microbiomes in Aquatic & Natural Ecosystems; (III) Microbes and Climate Resilience; and (IV) Microbiomes in Agriculture & Soil Health. A diverse program of invited talks, oral presentations, and posters showcased emerging research across diverse ecosystems and application domains. ISME Africa 2 extended its impact beyond the conference itself by organizing a fully funded, hands-on training workshop on bacterial genome sequencing. This advanced course brought together 20 early-career scientists, 10 from different African countries and 10 from Moroccan institutions, providing intensive capacity building and strengthening genomics expertise across the continent. Together, these efforts position ISME Africa as a catalyst for scientific excellence, collaboration, and sustainable microbiome research in Africa. Beyond these metrics, the meeting served as a convergence point for identifying shared priorities across regions and disciplines. Discussions consistently emphasized the need for harmonized methodologies, African-led research governance, stronger intra-continental collaboration, and closer integration of microbiome science with policy and One Health frameworks. In this sense, ISME Africa 2 functioned not only as a scientific forum but as a catalyst for shaping a coordinated continental research agenda.

### Past and future African contributions to microbial ecology

Advances in genomics and microbiome science increasingly rely on high-quality reference genomes and large-scale microbial datasets. Yet, most existing resources are derived from samples collected in Europe, North America, and East Asia, leaving African microbial biodiversity across ecosystems, agriculture, and human populations profoundly underrepresented. Reference genomes specific to African populations and ecosystems therefore have significant potential. As Africa is the continent where the earliest humans evolved, its human population is among the most genetically diverse; African lifestyles are also highly diverse, ranging from traditional farming communities to pastoralists to hunter-gatherers [11, 12]. The African human microbiome offers a unique opportunity to study microbiome–host co-evolution and holds substantial potential for fundamental research oriented toward One Health integration. Furthermore, Africa harbors an entire floristic region (the Cape floristic region) [13], a distinct biogeographic realm (the Afrotropical realm) [14], and two putative centers of plant domestication (the Sahel and Ethiopia) [15], making it a major, nonredundant center of interest for plant genomics and plant-associated microbiomes.

Particularly for nitrogen-fixating rhizobia, important discoveries have been made in Africa, including the identification of *Burkholderia* as a symbiotic bacterium and evidence of symbiosis occurring without *nodABC* genes [16]. A variety of drought-resistant crops are also found in Africa, including sorghum, which is a model monocot for drought research [17], and cowpea, a widely used drought-tolerant nitrogen-fixing legume, both of which originate from Africa [18]. We therefore expect Africa to provide crucial innovations in microbiome-based technologies for drought resistance. Africa also hosts a wide diversity of traditional, low-input farming and desert-farming systems. It is estimated that 65% of Africans engage in livestock farming in systems highly dependent on rainfall and climate variability [19], making the continent a major focus for One Health research at the human–animal–environment interface in the context of climate change.

African agroecosystems have often been characterized as low productivity; however, they are currently undergoing intensification, representing an invaluable opportunity to develop and study sustainable intensification strategies. Microorganisms indeed play a key role in this transformation, particularly through stress-resistant rhizobia and arbuscular mycorrhizal fungi [20, 21]. This transition provides a clear opportunity to operationalize a “From Africa, For Africa” framework, in which continentally generated microbiome knowledge forms the basis for locally relevant innovations and biotechnological applications.

Africa may be the continent most affected by pandemics (HIV, Ebola, COVID-19), and as such, genomic monitoring of human pathogens is a major priority. African institutions are now increasingly able to generate local genomic data in the medical domain [22]. Recent successes include the identification of the source of an Ebola outbreak in Guinea [23] and the “world genomic speed record” for diagnosing an Ebola infection in patients in Uganda [24]. South Africa was the second country globally to implement a national genomic surveillance COVID-19 program, which contributed to the launch of the Climate Amplified Diseases and Epidemics initiative [25]. Africa can now be considered a leader in expertise and infrastructure related to genomic pathogen surveillance.

Beyond classical pathogen surveillance, emerging work on microbiome analysis and environmental metagenomics is increasingly revealing the importance of antimicrobial resistance (AMR) dissemination across aquatic systems, where water networks act as major reservoirs and transmission corridors for resistance genes. Recent work in South African urban wastewater–river continuum has demonstrated that extracellular DNA represents a stable and ecologically distinct reservoir of high-risk mobile AMR genes, including those conferring resistance to last-resort antibiotics, and that both wastewater effluents and downstream rivers can act as cumulative sources of resistance dissemination [26]. These findings highlight how wastewater, river systems, and downstream reuse pathways facilitate the persistence and spread of AMR across interconnected environments, linking human activity, agriculture, and natural ecosystems. This issue is particularly relevant in regions where treated wastewater is increasingly reused for irrigation and aquifer recharge, including across North Africa and the broader Middle East and North Africa (MENA) region. In Morocco, for example, national strategies are actively promoting the expansion of wastewater reuse for agricultural production and groundwater replenishment, further underscoring the need for integrated microbiome-based AMR surveillance frameworks. In this context, continent-wide microbiome monitoring provides a critical tool to track environmental AMR dynamics and inform One Health interventions across Africa and adjacent regions.

### Building on existing research initiatives

**Figure 2:**
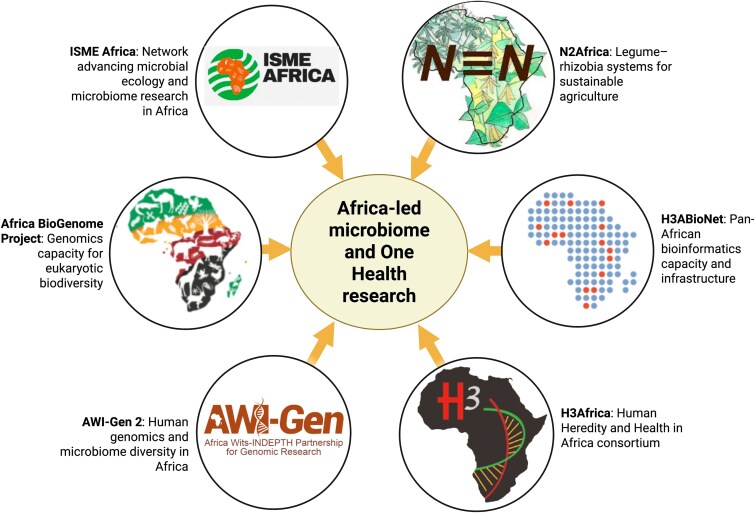
Convergent efforts to connect existing African initiatives around shared goals and common interests, fostering complementarity rather than competition to advance Africa-led microbial ecology, microbiome research, and One Health across health, agriculture, and environmental systems. Initiatives include ISME Africa (through the ISME Office), A2Africa (funded by the Bill & Melinda Gates Foundation), the AfricaBP, funded by the Wellcome Trust and African partner institutions, AWI-Gen 2 (supported by H3Africa, the U.S. National Institutes of Health, and the Wellcome Trust), H3Africa (funded by the NIH and Wellcome Trust), and H3ABioNet (supported by H3Africa and partner institutions). Collectively, these programs provide funding, training, infrastructure, and collaborative platforms to strengthen continental research capacity and coordination.

Initiatives such as the AWI-Gen 2 Microbiome Project and the AfricaBP provide proof-of-concept models for large-scale, population-representative studies integrating genomic and microbiome data collection (Fig. 2). Together, these initiatives signal a transition from fragmented, externally driven studies toward more coordinated, African-led research efforts, although this transition remains incomplete. AWI-Gen 2, supported through the Human Heredity and Health in Africa (H3Africa) program with funding from the U.S. National Institutes of Health (NIH) and the Wellcome Trust, and AfricaBP, backed by a consortium of African institutions and international partners, demonstrate the feasibility of coordinated, continent-wide research platforms. Beyond data generation, these initiatives prioritize training African scientists in sequencing technologies, bioinformatics, and advanced data analysis, thereby strengthening local scientific leadership [6, 9].

Complementing the continental coordination, data generation, and capacity-building efforts led by AWI-Gen 2 and AfricaBP, the African Genome Center at the University Mohammed VI Polytechnic (UM6P) serves as a regional hub for microbiome and climate-resilience research, coordinating multi-institutional projects across Africa that map microbiomes of agroecosystems and native, endemic, and endangered plants, integrating genomics, metagenomics, and environmental data to generate Africa-specific reference microbiomes and strengthen cross-border collaboration. At a broader scale, the African microbiome mapping initiative and the “One Continent: One Vision” framework provide conceptual coordination for continent-wide microbiome research (Fig. 2) [1, 27]. These early efforts demonstrate both the feasibility and urgency of scaling coordinated microbiome research across Africa, moving from isolated projects toward integrated, continent-wide platforms.

Several key lessons consistent with global microbiome perspectives emerge from early initiatives. First, population representativeness is critical, requiring inclusion of urban, rural, and indigenous populations to capture Africa’s ecological, cultural, and biological diversity. Second, regional hubs amplify impact by enabling multi-country collaborations, training, and data integration. Third, robust digital infrastructure and harmonized bioinformatics pipelines are critical for implementing FAIR (Findable, Accessible, Interoperable, and Reusable) data principles, which ensure that microbiome datasets can be shared, compared, and reused across institutions and countries. Despite these advances, bioinformatics and computational capacity remain a major bottleneck for scaling microbiome research across the continent. The rapid expansion of sequencing efforts has outpaced capabilities for data processing, integration, and interpretation, limiting the translation of raw data into biological insight and actionable outcomes. Addressing this gap will require sustained investment in high-performance computing, cloud-based platforms, and standardized, reproducible analysis pipelines capable of handling large-scale metagenomic and multi-omics data. Strengthening these capabilities is essential not only for ensuring data quality and comparability but also to enable African researchers to lead data-driven discovery and innovation. Collectively, these initiatives provide a clear blueprint for continental coordination, demonstrating that Africa can generate high-quality, locally relevant microbiome datasets while simultaneously strengthening scientific capacity, research leadership, and scientific sovereignty.

### Establishing pan-African research networks

Fragmentation remains one of the most persistent barriers to microbiome research in Africa. Intra-continental collaboration remains limited, while leadership and knowledge exchange are often dominated by senior researchers based outside the continent [3, 4, 28]. Existing regional initiatives, including the Arab Research and Innovation Co-Funded Alliances (ARICA), support multi-country partnerships across the MENA region through coordinated co-funding mechanisms, but these efforts remain geographically concentrated and do not yet extend across the broader African research landscape. This structural imbalance constrains inclusivity, sustainability, and contextual relevance of microbiome research.

Strategic pan-African research networks are required alongside sustained capacity development. Initiatives such as H3Africa [29] and Afrinom [28] exemplify the transformative potential of multi-country networks that integrate capacity building with coordinated research efforts. These networks have enabled bioinformatics training, harmonized sample collection, and shared data platforms across countries. By connecting geographically dispersed researchers, such networks reduce duplication, strengthen leadership, and improve coordination (Fig. 1).

Long-term sustainability of these networks depends on equitable partnerships. African researchers must serve as principal investigators and intellectual leaders, setting research agendas that reflect African microbiome priorities. Without such coordinated networks, microbiome research in Africa will remain fragmented, limiting its ability to generate scalable and globally relevant insights. Funding models must prioritize intra-African collaboration while promoting fair authorship practices and equitable data ownership. In addition, multi-site training programs, regional centers of excellence, and structured mentoring frameworks are essential fir sustaining these networks and cultivating the next generation of African scientific leaders.

Regional initiatives such as ARICA provide important groundwork within MENA; however, inclusive continent-wide pan-African networks remain essential for scaling microbiome research and ensuring long-term scientific and societal impact.

### Developing flagship research programs

We propose that a limited number of high-impact, continental flagship programs should anchor microbial ecology research across Africa. To translate emerging microbiome efforts into sustained impact, Africa needs coordinated, high-visibility research programs to guide investment and set scientific priorities. Developing such flagship programs (Fig. 1) is essential to focus attention and resources on the continent’s highest-priority microbiome challenges. In this context, One Health integration plays a central role by linking human, animal, and environmental microbiomes to better understand pathogen dynamics, nutritional impacts, and ecosystem services [1].

Antimicrobial resistance surveillance is a critical priority, with genomic monitoring of pathogens increasingly supported by initiatives such as the Africa Centers for Disease Control and Prevention (Africa CDC) and the Pathogen Genomics Initiative [30, 31]. As AMR is linked to water quality and sanitation, metagenomic approaches are required to assess the presence of resistance genes in water reservoirs and wastewater treatment plants, positioning microbial ecology at the intersection of water management and AMR control.

Precision medicine approaches leveraging Africa-specific microbiomes can guide interventions targeting undernutrition, infectious diseases, and noncommunicable conditions [4, 32]. African populations harbor the highest levels of human genetic diversity globally; consequently, findings derived primarily from European and North American cohorts may not be directly transferable in precision medicine contexts. Initiatives such as the African Collaborative Center for Microbiome and Genomics Research based in Nigeria [33], the CSIR Microbiome Mapping Initiative, the African Microbiome Project, and the AWI-Gen, several of which are integrated within the H3Africa Consortium, are advancing this agenda. These priority areas not only address pressing regional challenges but also position Africa at the forefront of globally relevant microbiome research and innovation.

Food security and climate-resilient agriculture have been mainstreamed at the national level by countries such as Kenya through the Kenya Climate-Smart Agriculture Project and at the international level through initiatives such as the Alliance for a Green Revolution in Africa and the International Fund for Agricultural Development. Their aim to increase agricultural productivity while strengthening resilience to climate change relies in part on understanding and engineering soil microbiome responses to stress. However, such studies are inherently long term, often requiring several successive years to yield robust and predictable results [34, 35].

Beyond health-focused applications, flagship programs should prioritize large-scale, interdisciplinary mapping of microbiomes across Africa’s agroecosystems, natural environments, and underexplored habitats. Although important efforts have been undertaken in specific regions and ecosystems, including desert microbiome studies and localized environmental surveys, these initiatives remain geographically and ecologically fragmented, covering only parts of the continent [36]. A coordinated, pan-African strategy is therefore needed.

Systematic characterization of microbiomes associated with crops, soils, rangelands, deserts, wetlands, forests, freshwater systems, extreme habitats, and marine ecosystems would not only establish critical ecological baselines and uncover context-specific microbial functions relevant to food security, climate resilience, and ecosystem restoration but also position Africa within the emerging global movement to recognize and safeguard microbial diversity as a vital biological component of Earth’s natural heritage [36–38]. Achieving these objectives will require harmonized and standardized protocols to ensure cross-site comparability, longitudinal sampling frameworks to capture temporal dynamics, and the systematic integration of environmental metadata to link microbial patterns with their ecological drivers, approaches consistent with emerging global strategies to safeguard microbial biodiversity, including initiatives within the IUCN Species Survival Commission’s Microbial Conservation Specialist Group [39].

Recent continent-scale evidence further reinforces this need [40]. A large environmental DNA amplicon sequencing survey across 32 African countries revealed pronounced spatial heterogeneity in soil fungal diversity, with clear hotspots in savannas and tropical forests and coldspots in arid regions. Precipitation and latitudinal gradients emerged as dominant drivers of fungal alpha diversity, whereas climate and soil properties structured beta-diversity patterns. These findings provide the first high-resolution continental map of African soil fungal richness, highlighting both the vast, underexplored diversity of soil microbiomes and the urgency of integrating these datasets into coordinated, African-led research frameworks and conservation strategies [40].

A complementary large-scale microbiome mapping and ecosystem characterization efforts, another priority is the establishment of African-owned microbial collections and biobanks to preserve beneficial and novel microbes and genetic materials for agricultural and environmental applications. Although microbial collections exist in Africa, they are largely concentrated in South Africa, highlighting the need for additional facilities in North, East, and West Africa. According to the World Federation for Culture Collections (https://www.wfcc.info/membership/memberlist), only three collections in Africa are currently listed, two in South Africa and one in Morocco. Expanding this infrastructure would enable the development of locally adapted biocontrol products rather than relying on imports from other continents and would also create valuable economic opportunities. Their implementation could build on the expertise of established South African collections, not only in isolation and culturing workflows but also in strain barcoding and patenting.

Another promising area for agricultural applications of microbiome research is the preservation and use of whole microbiomes. Although this remains an emerging field, its integration of metagenomics-based approaches with microbial collection strategies makes it a particularly compelling area for future development. These flagship programs are most effective when research is integrated with capacity building and policy engagement, ensuring that findings and products are actionable and locally relevant. For instance, linking AMR surveillance to national health systems supports public health decision-making, whereas characterization of soil microbiomes informs strategies to support climate-resilient farming. Taken together, such flagship programs provide a mechanism for aligning scientific discovery with implementation, ensuring that microbiome research translates into tangible societal and environmental benefits.

### Integrating capacity building, policy, and open science

Integrating capacity building, policy engagement, and open science is essential for the sustainable development of microbiome research in Africa. Although recent initiatives have expanded sequencing and bioinformatics capabilities, persistent constraints, including uneven infrastructure distribution, high operational costs, and reliance on external facilities, continue to limit scalability and data sovereignty [3, 41]. A key strategy for addressing these challenges is developing of regional hubs that provide coordinated training and research support [2]. Networks like H3ABioNet illustrate how such hubs can strengthen technical expertise (Fig. 2), foster cross-country collaboration, retain scientific talent within the continent, and support standardized workflows and shared resources.

Alongside strengthening regional training and research infrastructure, integrating microbiome science into national and regional policy frameworks. Advocacy for increased government investment is critical, as current funding remains predominantly external, with South Africa as an exception [3]. Aligning microbiome research with public health, agricultural, and environmental priorities can strengthen political support and ensure that research outcomes directly inform decision-making processes.

Open science practices further reinforce this ecosystem by enabling transparency, data reuse, and collaboration. Adoption of FAIR data principles, standardized metadata, and Africa-centered repositories, such as emerging microbiome data portals, enhances reproducibility, global visibility, and equitable participation in microbiome research. Together, coordinated capacity building, policy integration, and open science provide a foundation for scaling microbiome research strengthening African scientific leadership and data sovereignty [6, 42]. These components collectively define the foundation of a self-sustaining African microbial ecology research ecosystem, reducing long-term dependence on external infrastructure, funding, and leadership.

## Discussion

Microbial ecology research in Africa is at a critical inflection point. Recent initiatives and growing continental dialogue signal a transition from fragmented efforts toward more coordinated, African-led systems. At the same time, Africa’s exceptional ecological, demographic, and epidemiological diversity remains an underutilized resource for advancing global microbiome science. The persistent underrepresentation of African microbiomes in global datasets therefore reflects not only a gap but a strategic opportunity to expand and rebalance the field.

Africa encompasses highly heterogeneous research ecosystems, with uneven distribution of infrastructure, capacity, and investment across regions. Large-scale initiatives such as AWI-Gen 2 and the AfricaBP demonstrate that population-representative studies, coordinated data generation, and regional training are feasible within African contexts. Pathogen genomics has emerged as a domain of strength, with African institutions increasingly recognized for their expertise in genomic surveillance. Nevertheless, these advances also highlight a broader structural imbalance in global microbiome research as knowledge production remaining geographically concentrated, reinforcing disparities in scientific leadership, data ownership, and research priorities.

Addressing the uneven distribution of infrastructure and investment, fragmented research coordination, and persistent disparities in scientific leadership and data ownership requires a shift from externally driven, fragmented projects toward a coordinated, African-led research ecosystem that integrates infrastructure, data systems, and policy across One Health domains. While full continental integration remains a long-term objective, regional collaboration hubs in West, East, Southern, and North Africa represent practical and scalable intermediate steps. A multi-level framework combining continental coordination with strong regional networks will be essential to accommodate diversity while enabling harmonization and scalability.

Flagship research programs focused on One Health, AMR, food security, and precision medicine are well aligned with both African development priorities and global challenges. However, microbiome studies targeting the highest-burden conditions remain limited. Strengthening impact will require closer alignment between research agendas and local needs, as well as greater leadership by African scientists in study design, governance, and interpretation. Sustaining and scaling these efforts will require deliberate investment in pan-African research networks, strengthening sequencing and bioinformatics infrastructure, and the development of policies that integrate microbiome research into national health and agricultural strategies. Finally, it is essential to reinforce training and mentorship for early-career African scientists to build long-term research capacity.

A transformative priority is the development of an integrated Pan-African Microbiome Commons, a network-of-networks linking regional hubs, shared data infrastructures, and coordinated training systems. Such a framework would enable harmonized sampling strategies, equitable data ownership, distributed analytical capacity, and cross-border training pipelines. Critically, it would create sustained opportunities for African scientists to lead multi-country projects, co-develop research agendas, and engage equitably in global consortia.

Africa is uniquely positioned not only to generate locally relevant microbiome data but to reshape global microbiome science. Advancing more inclusive, representative, and ecologically comprehensive models aligned with One Health and planetary health principles, the continent has the potential to shift the center of gravity of microbiome research toward a more equitable, globally relevant paradigm.

## Conclusion and future directions

The lack of African reference data remains a major bottleneck to advancing health, agriculture, and environmental sustainability across the continent and globally. Recent flagship programs and regional capacity-building efforts demonstrate meaningful progress, particularly in generating population-representative data, strengthening technical expertise, and fostering collaborative networks. Although these advances remain unevenly distributed, reflecting disparities in infrastructure, investment, and research capacity, they collectively signal growing momentum toward a more integrated, African-led microbiome research landscape. The next phase is the development of a Pan-African Microbiome Commons, envisioned as a continent-wide network-of-networks integrating regional hubs, shared data infrastructures, coordinated sampling frameworks, and harmonized bioinformatics pipelines. Within this framework, bioinformatics must be positioned as a foundational pillar. Beyond data generation, the Commons should incorporate distributed computational infrastructure, interoperable data platforms, and shared analytical workflows to enable cross-country harmonization and real-time collaboration. Leveraging advances in machine learning and artificial intelligence will further enhance the identification of microbial patterns, prediction of ecosystem responses, and support for precision interventions in health, agriculture, and environmental management. Embedding scalable, Africa-centered computational ecosystems within this framework will enable transition from fragmented data generation to integrated, high-impact microbiome science. Such a Commons would move beyond traditional collaboration models by enabling joint data ownership, interoperable platforms, and distributed analytical capacity, while embedding training, mobility, and leadership development opportunities for African scientists. By connecting institutions, ecosystems, and disciplines, it offers a practical and scalable pathway to transform fragmented efforts into a cohesive and sustainable research system.

Looking forward, the success of this model will depend on strong alignment between open science principles, equitable partnerships, and policy integration. Embedding microbiome research within national and regional strategies will be essential to ensure that scientific outputs translate into actionable outcomes and impact in public health, agriculture, and environmental management. At the same time, expanding multi-site training programs, continental consortia, and shared infrastructure will be critical for building long-term capacity and retaining scientific talent within Africa. In this context, the Pan-African Microbiome Commons can serve not only as a scientific platform but also as a governance model that promotes data sovereignty, equitable authorship, and African leadership in global research initiatives.

Finally, scaling open science initiatives and FAIR-compliant, Africa-centered data platforms through such a Commons will enhance the accessibility, reuse, and global visibility of African microbiome datasets while ensuring appropriate data governance and benefit-sharing. By coordinating investment in research networks, capacity building, policy integration, and open science, Africa can transform fragmented efforts into cohesive, continent-wide One Health initiatives, positioning African researchers as leaders in microbiome science and microbial ecology.

## Data Availability

Data sharing is not applicable to this article.
